# 石蜡包埋组织中组蛋白的提取分离和翻译后修饰的定量分析

**DOI:** 10.3724/SP.J.1123.2021.06018

**Published:** 2021-10-08

**Authors:** Shanshan TIAN, Ranran LIU, Xiaolong QIAN, Xiaojing GUO, Kai ZHANG

**Affiliations:** 1.天津医科大学基础医学院, 天津市医学表观遗传学重点实验室, 天津 300070; 1. School of Basic Medical Sciences, Tianjin Medical University, Tianjin Key Lab for Medical Epigenetics, Tianjin 300070, China; 2.天津医科大学肿瘤医院乳腺病理研究室, 教育部乳腺癌防治重点实验室, 国家肿瘤临床医学研究中心, 天津 300060; 2. Department of Breast Pathology and Lab, Tianjin Medical University Cancer Institute and Hospital, Key Laboratory of Breast Cancer Prevention and Therapy, Ministry of Education; National Clinical Research Center of Cancer; Tianjin 300060, China

**Keywords:** 高效液相色谱-串联质谱, 组蛋白翻译后修饰, 临床组织样本, 石蜡包埋, 乳腺癌, high performance liquid chromatography-tandem mass spectrometry (HPLC-MS/MS), histone post-translational modifications (HPTMs), clinical tissues, paraffin-embedded, breast cancer

## Abstract

组蛋白翻译后修饰(HPTMs)参与基因转录调控,其异常与肿瘤等重大疾病的发生发展密切相关。石蜡包埋组织是当前疾病研究的重要样本资源,对肿瘤机制和标志物研究具有重要意义。目前基于质谱的蛋白质组学技术已成为HPTMs分析的有力工具,而针对福尔马林固定石蜡包埋(FFPE)组织样品的HPTMs分析还十分有限。该研究发展了一种基于高效液相色谱-串联质谱的FFPE组织样本HPTMs分离分析新方法。通过研究并优化组蛋白的提取策略,建立了FFPE组织样本脱蜡水化处理、蛋白质提取与聚丙烯酰胺凝胶电泳分离相结合的组蛋白提取和分离方法。通过研究FFPE切片数量、组蛋白化学衍生化方法等对组蛋白鉴定的影响,确定了组蛋白处理的具体步骤。通过HPLC分离结合非依赖性采集模式的质谱分析,鉴定了组蛋白修饰的类型、位点和丰度。最后,将优化的实验方法应用于FFPE临床样本的HPTMs分析,鉴定了2例人乳腺浸润性癌和癌旁正常组织的HPTMs图谱,均获得了100种以上的不同组蛋白修饰形式的多肽。定量分析了他们的差异性水平,通过主成分降维分析,发现浸润性癌和癌旁正常组织之间组蛋白修饰丰度存在明显的差异,且差异性具有一定的规律,特别是涉及转录调控的组蛋白修饰与乳腺癌的预后和治疗靶点具有相关性,进而探讨了乳腺癌中异常HPTMs的生物学意义。该研究对临床石蜡样本中组蛋白修饰的分离分析以及肿瘤表观遗传标志物的检测进行了有益的探索。

## Abstract (en)

Histone post-translational modifications (HPTMs) have been believed to play crucial roles in the regulation of gene transcription. Thus, aberrant modification of histone can contribute to the occurrence and development of diseases such as tumors. To date, formalin fixed paraffin-embedded (FFPE) clinical tissues are believed to be one of the most valuable sample resources in the study of human diseases. Therefore, it is of great significance to reveal the molecular mechanism of cancer and discover the markers of tumor. Proteomics, based on high performance liquid chromatograph-tandem mass spectrometry (HPLC-MS/MS), has become a powerful tool for HPTM identification. However, HPTM analysis of FFPE samples is yet to be explored; it has not been reported in China to our best knowledge. In this study, a new method based on HPLC-MS/MS was developed for the extraction and separation of histone proteins and analysis and quantification of HPTMs in FFPE tissues. First, the strategy for the extraction and separation of histone proteins from FFPE samples were optimized. After comparing the extraction efficiency of two different methods, which include the acid extraction of histone and extraction of total protein, a novel method was developed for histone extraction, separation, and HPTMs analysis by integrating dewaxed hydration treatment of FFPE tissues with protein extraction and sodium dodecyl sulfate-polyacrylamide gel electrophoresis (SDS-PAGE) separation. Furthermore, the effects of operation parameters on histone extraction and HPTM identification were investigated, including number of paraffin embedded sections and chemical derivation of histone proteins. Thereafter, the identification and quantification of HPTMs were performed using reversed-phase HPLC-MS/MS in data independent acquisition (DIA) mode. Finally, the optimized methods were applied to quantitative analysis of HPTMs in FFPE tissues. A variety of HPTMs were identified; they included lysine methylation, acetylation, crotonylation, etc. Therefore, the spectrum of HPTMs on global level was set for human breast cancer and paracancerous tissues from two cases of FFPE clinical tissues. The sites holding differential HPTM level in cancer and paracancerous tissues were further obtained by quantifying the individual HPTMs. Thus, the relationship between HPTM level and tumor was discussed. Abnormal HPTM sites were characterized using cluster analysis, thus their similar tendency was found. For example, abnormal HPTM sites such as H3K9me3, H3K9ac, and H3K27me3 in cancers are associated with epigenetic regulation. It indicated that different epigenetic events might occur in cancer and paracancerous tissues. Interestingly, we found that the level of H4K20me3 were both significantly down-regulated in the two cancer tissues. HPTM had been thought to be a potential biomarker in breast cancer; therefore, these positive results indicated that our method is effective for HPTMs of clinical FFPE samples. Our study provides a useful tool for the isolation and analysis of HPTMs in clinical FFPE samples, showing the potential for the detection of epigenetic biomarker in cancer.

组蛋白(histone)是真核生物核小体的主要蛋白质,包括核心组蛋白H2A、H2B、H3、H4以及H1,其翻译后修饰(HPTMs)是一类重要的表观遗传调控因子,在不同生理、病理条件下的基因表达调控中扮演重要角色^[1]^。近年来,随着蛋白质分离富集技术的快速发展,生物质谱扫描速度、分辨率和灵敏度的不断提高,越来越多的新型组蛋白翻译后修饰被相继发现^[2,3]^,极大地拓展了人们对于组蛋白密码(histone code)的认识。大量研究^[4]^表明,组蛋白翻译后修饰的异常表达或定位,与肿瘤的发生发展密切相关,因此,全面系统地分析正常组织和病变组织中组蛋白翻译后修饰位点与修饰水平,对于理解组蛋白修饰功能、揭示肿瘤发生机制和标志物,以及寻找药物靶标有着十分重要的意义^[5]^。

当前对于临床样本的HPTMs研究,往往借助基于抗体的免疫组化等手段,然而这些方法有其自身的局限性,如抗体种类少、难以进行HPTMs系统分析、无法检测组合修饰形式等^[6]^。而基于质谱的蛋白质组学技术,是解决上述问题的有力工具^[7]^,并已应用于细胞系和动物组织中表观遗传修饰的分析研究中^[8,9]^。然而体外培养细胞组蛋白翻译后修饰水平的差异并不能够完全反映体内细胞的真实状况,因此从临床病理组织中提取组蛋白并分析其翻译后修饰水平有很重要的意义^[10]^。中性福尔马林固定石蜡包埋(FFPE)是当前临床病理组织的常规储存方式,能够长期保存并维持原始的细胞形态和组织结构。临床石蜡样本往往包含有丰富的病理资料和临床信息,是人类疾病研究中非常宝贵的样本资源^[11]^。目前,人类临床样本的蛋白质组学分析已取得显著进展^[12,13]^,但是HPTMs分析方法与应用研究仍十分有限^[14]^,特别是石蜡包埋组织样品的HPTMs质谱定量分析国内尚未见报道。

本研究发展了一种基于高效液相色谱-串联质谱的石蜡包埋样本组蛋白翻译后修饰分离分析方法。首先,设计了两种组蛋白提取方法,一种是将石蜡包埋组织脱蜡水化处理后与组蛋白酸提取方法相结合^[15]^,另一种是将石蜡包埋组织脱蜡水化处理后与全蛋白提取方法相结合,借助于BCA蛋白浓度检测和聚丙烯酰胺凝胶电泳分离技术对组蛋白提取量进行评价,进而研究石蜡包埋切片的数量、组蛋白化学衍生化对鉴定的影响。采用丙酸酐化学衍生化策略对组蛋白裸露的赖氨酸和多肽的N端分别进行胶内和溶液内标记反应,避免由于组蛋白富含赖氨酸和精氨酸引起的酶切多肽过短的问题,同时增加多肽疏水性,利于分离分析。最后,将优化的组蛋白提取方法与HPTMs分析实验流程应用于人乳腺癌石蜡包埋临床样本中组蛋白翻译后修饰的定性定量分析中,并借助于Proteome Discoverer、EpiProfile等软件^[16]^,对质谱数据进行检索与生物信息学分析。该方法能够实现石蜡包埋临床样本中甲基化、乙酰化等组蛋白翻译后修饰的定性定量分析,可帮助筛选不同病人之间的HPTMs差异。这一研究有助于临床石蜡样本组蛋白修饰分离分析研究的深入开展。

## 1 实验部分

### 1.1 仪器、试剂与材料

低温超速离心机、EASY-nLC 1000 LC-MS/MS纳升级超高效液相色谱-串联质谱Q-Exactive质谱仪、酶标仪、真空旋干浓缩仪均购自Thermo Fisher Scientific公司(美国);超声波破碎仪购自Sonics公司(美国);恒温金属浴购自Eppendorf公司(德国)。

BCA蛋白定量试剂盒(BCA Protein Assay Kit)、HPLC级乙腈(ACN)、HPLC级水、甲酸(FA)均购自Thermo Fisher Scientific公司(美国);三氟乙酸(TFA)、丙酸酐、氨水购自Sigma-Aldrich公司(美国);碳酸氢铵(NH_4_HCO_3_)、十二烷基硫酸钠(SDS)、乙基苯基聚乙二醇(NP-40)、三羟甲基氨基甲烷(Tris)、氯化钠、考马斯亮蓝、苯甲基磺酰氟(PMSF)等购自上海生工生物工程有限公司;去乙酰化酶抑制剂混合物(Deacetylase Inhibitor Cocktail)购自上海碧云天生物技术有限公司;全蛋白酶抑制剂(Protease Inhibitor Cocktail)购自上海罗氏制药有限公司;三氯乙酸(TCA)购自上海阿拉丁生化科技股份有限公司;二甲苯、乙醇、丙酮购自天津市化学试剂供销公司;胰蛋白酶(trypsin)购自Promega公司(美国); Micro-C18 Zip-Tip除盐柱购自Millipore公司(美国);去离子水由Millipore纯水仪制备;化学试剂纯度为分析级或HPLC级。

### 1.2 实验方法

1.2.1 石蜡包埋组织中组蛋白的提取

结合光学显微镜观察结果,用手术刀片分别刮取苏木精-伊红染色石蜡包埋组织切片的癌组织(cancer tissues, C)和癌旁正常组织(normal tissues, N),依次加入二甲苯脱蜡和梯度乙醇水溶液(95%、70%、50%、20%和超纯水)水化组织,于4 ℃以13600 r/min离心3 min,弃上清。加入200 μL SDS裂解液(100 mmol/L Tris、100 mmol/L NaCl、69.4 mmol/L SDS, pH 8.0)裂解组织,超声破碎溶解后进行热修复过程(在95 ℃下孵育45 min,然后在65 ℃下孵育4 h),以13600 r/min离心5 min,获得全蛋白裂解液。采用BCA法测量蛋白质浓度,取40 μg全蛋白裂解液上样,并采用SDS-聚丙烯酰胺凝胶电泳(PAGE)的方法(12%的分离胶和6%的浓缩胶)进行蛋白质电泳分离,电泳完成后用考马斯亮蓝进行染色30 min,随后将凝胶脱色可获得清晰的组蛋白条带。

对于酸提取法,脱蜡、水化并离心后,加入200 μL NIB裂解液(15 mmol/L Tris、60 mmol/L KCl、15 mmol/L NaCl、5 mmol/L MgCl_2_、1 mmol/L CaCl_2_、250 mmol/L蔗糖(sucrose)、终体积分数1%(v/v)NP-40, pH 7.5)裂解组织,超声破碎溶解后进行热修复过程(在95 ℃下孵育45 min,然后在65 ℃下孵育4 h),以13600 r/min离心5 min,弃上清。加入400 μL的0.2 mol/L H_2_SO_4_溶液溶解。加入终体积分数25%(v/v)TCA沉淀组蛋白,经预冷丙酮清洗2次后,获得组蛋白沉淀物。用水溶液将沉淀物溶解后全部上样,再次采用SDS-PAGE方法(12%的分离胶和6%的浓缩胶)进行蛋白质电泳分离,电泳完成后用考马斯亮蓝进行染色30 min,随后将凝胶脱色可获得组蛋白条带。

1.2.2 组蛋白的胶内化学衍生化与酶解

将考马斯亮蓝染色的组蛋白条带切碎并转移至1.5 mL离心管中,依次加入1 mL 25 mmol/L NH_4_HCO_3_ 50%(v/v)乙醇水溶液、100 mmol/L NH_4_HCO_3_溶液和ACN振荡10 min。随后进行组蛋白胶内的丙酸酐衍生化,加入200 μL标记试剂丙酸酐-乙腈(1;3, v/v)和200 μL 100 mmol/L NH_4_HCO_3_,并采用纯氨水调节溶液pH值至8,于37 ℃反应15 min。弃上清,重复2次衍生化操作。按照胰蛋白酶与蛋白质的质量比为1;25加入胰蛋白酶,于37 ℃水浴酶解过夜。依次加入ACN-H_2_O-TFA(10;9;1, v/v/v)、ACN-H_2_O-TFA(750;249;1, v/v/v)和ACN,提取酶解多肽并真空旋干。加入50 μL 100 mmol/L NH_4_HCO_3_溶解多肽,进行溶液内多肽N端的丙酸酐衍生化,加入15 μL标记试剂丙酸酐-乙腈(1;3, v/v),调节溶液pH值至8,于37 ℃反应15 min。重复2次衍生化操作,以保证反应标记效率。

1.2.3 LC-MS/MS分析

样品经Micro-C18 Zip-Tip柱除盐后,加入7 μL含0.1%(v/v)FA的水溶液溶解,从中取5 μL注射到Nano-LC系统(EASY-nLC 1000),样品通过Acclaim pepMap RSLC C18反相毛细管柱(15 cm×75 μm, 3 μm,美国Thermo Fisher Scientific公司)进行反相色谱分离,流动相A和B分别为含0.1%(v/v)FA的水溶液和含0.1%(v/v)FA的乙腈溶液,分离后的组分经电喷雾电离(ESI)后,进入到Orbitrap Q-Exactive质谱系统进行分析。

利用数据依赖采集(data-dependent acquisition, DDA)模式进行分析时使用液相梯度洗脱程序,具体为0~16 min, 5%B~13%B; 16~51 min, 13%B~28%B; 51~66 min, 28%B~45%B; 66~67 min, 45%B~100%B; 67~75 min, 100%B。电喷雾电压设置2.4 kV,一级谱图扫描时,自动增益控制(AGC)设置为1×10^6^扫描范围为*m/z* 350~1800,分辨率为70000,采用高能碰撞解离(HCD)方式对峰度排序前15的2价多肽依次进行选择并碰撞破碎,归一化碰撞能量设为27%,二级谱图的扫描分辨率设为17500, AGC设为5×10^4^,隔离窗口(*m/z*)为2.2,动态排除时间设为15 s。

实验利用数据非依赖采集(data-independent acquisition, DIA)模式进行分析时使用液相梯度洗脱程序,具体为0~55 min, 6%B~45%B; 55~60 min, 45%B~100%B; 60~70 min, 100%B。电压设置2.4 kV,一级谱图扫描时,AGC设为1×10^6^,扫描的范围为*m/z* 350~1100,分辨率为35000,采用高能碰撞解离,归一化碰撞能量设为27%,二级谱图的扫描分辨率设为17500,隔离窗口(*m/z*)为50, AGC设为5×10^5^。

1.2.4 数据分析

组蛋白翻译后修饰的定性鉴定采用Proteome Discoverer (PD,版本1.4)软件对DDA模式下的数据进行检索,数据库为Uniprot-Histone Human。检索参数设置为最多允许5个胰蛋白酶漏切位点,每个肽段最多修饰数设为5个,可变修饰包括赖氨酸乙酰化、单甲基化、二甲基化、三甲基化、琥珀酰化、巴豆酰化、二羟基异丁酰化、丙二酰化,假阳性率(FDR)≤0.01;前体离子和MS/MS允许误差范围分别设定为10^-6^(10 ppm)和0.02 Da。

组蛋白翻译后修饰定量分析:采用非标记质谱定量方式,使用EpiProfile 2.0软件对DIA模式下的数据进行检索^[16]^,根据*m/z*、保留时间等提取各个组蛋白酶解肽段的色谱峰面积,以提取的氨基酸序列相同的所有肽段(包括其所有修饰形式及未修饰形式)色谱峰下的总面积为100%,计算每个修饰肽段的相对百分比。对于相对分子质量相同的多肽,其相对比例通过不同质量二级碎片离子的峰强度进行辨别与计算。每个样品分别进行3次实验重复,对定量结果取平均值后,比较癌组织和癌旁正常组织组蛋白修饰的差异,并进行生物信息学分析。

### 1.3 伦理声明

2例中性福尔马林固定石蜡包埋乳腺癌组织(包括浸润性癌和癌旁正常组织)由天津医科大学肿瘤医院乳腺病理研究室提供,所有患者在样本被使用之前都签署了知情同意书,该研究项目得到天津医科大学肿瘤医院伦理委员会的批准。

## 2 结果与讨论

### 2.1 石蜡包埋组织中组蛋白的提取方法与条件优化

2.1.1 组蛋白提取策略与评价

组蛋白的提取往往采用酸提取的方法,即利用组蛋白富含赖氨酸和精氨酸等碱性氨基酸可以很好地在酸中溶解的特性,将胞浆蛋白、核内其他杂蛋白与碱性组蛋白进行分离,从而获取主要组蛋白成分(H1/H2A/H2B/H3/H4)。因此,设计了实验流程A(见图1),首先采用二甲苯和梯度的乙醇水溶液分别对石蜡包埋组织进行脱蜡和水化,然后利用NIB裂解液破坏细胞膜并进行热修复过程,最后利用硫酸提取获得组蛋白。

**图1:**
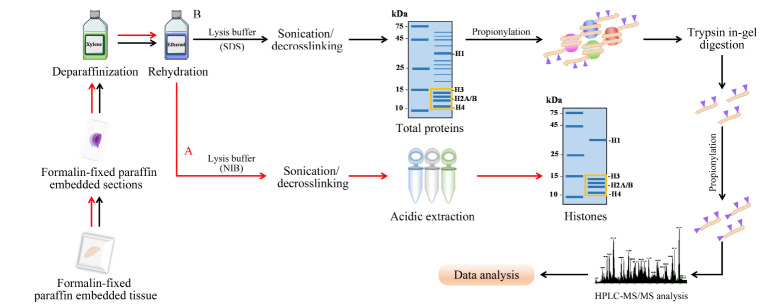
福尔马林固定石蜡包埋组织中组蛋白翻译后修饰定量分析流程图

通过BCA试剂盒对提取的组蛋白进行定量分析,4片10 μm(4×10 μm)石蜡包埋组织可提取约3 μg组蛋白样品,通过聚丙烯酰胺凝胶电泳分离进行表征,组蛋白条带较浅(图2a),提取产量较少,不利于后续组蛋白修饰的深度分析。这可能是因为此方法涉及去除胞浆蛋白、冰丙酮清洗等实验步骤,造成石蜡包埋样本组蛋白损失较多。考虑到SDS具有更强的裂解效果,我们同时设计了实验流程B(见图1),在对石蜡包埋组织进行脱蜡和水化后,加入SDS裂解液裂解组织同时进行热修复过程,获取全蛋白裂解液,经聚丙烯酰胺凝胶电泳分离获得组蛋白。通过BCA蛋白浓度检测,4片10 μm石蜡包埋组织可提取约200 μg全蛋白样品,借助聚丙烯酰胺凝胶电泳分离去除杂蛋白,可以获得清晰的组蛋白条带(见图2b)。因此,采用实验流程B进行下一步条件优化。

**图2:**
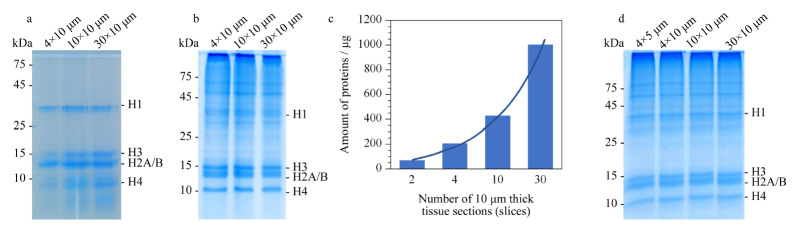
福尔马林固定石蜡样本组蛋白提取的评价和优化

2.1.2 组蛋白提取方法的条件优化

根据实验设计流程,分别对不同数量石蜡包埋切片的蛋白质提取量(见图2c)、组蛋白提取效果(见图2d)、组蛋白化学衍生化效率进行了考察。通过BCA蛋白浓度检测结果显示,随着切片数量的增加,蛋白质的提取量呈现倍数增长。2片10 μm切片即可提取约70 μg蛋白质含量,满足后续实验需要。4片10 μm切片可提取约200 μg蛋白质含量,可实现3次实验重复。取相同含量(40 μg)的蛋白质进行聚丙烯酰胺凝胶电泳分离,经考马斯亮蓝染色后,不同数量切片提取的组蛋白条带无明显差别。多肽N端衍生化使用10 μL标记试剂时,数据分析发现,组蛋白酶解多肽的N端标记效率偏低(约80%左右)。提高多肽N端衍生化标记试剂至15 μL时,组蛋白酶解多肽的N端标记效率可提高至95%以上。因此,组蛋白翻译后修饰定量分析可采用4片10 μm切片、多肽N端衍生化标记试剂15 μL的实验条件。

### 2.2 乳腺癌石蜡包埋临床样本中组蛋白翻译后修饰分析

2.2.1 样本选取与组蛋白提取

结合苏木精-伊红染色切片在光学显微镜下的观察结果,能够精准确定浸润性癌与癌旁正常组织的位置,减少组织纯度引起的结果误差。以苏木精-伊红染色切片中癌与癌旁正常组织的位置为参考,刮取4片10 μm未染色切片中的癌和癌旁正常组织(见图3a),加入SDS裂解液裂解组织并进行热修复过程,采用聚丙烯酰胺凝胶电泳对组蛋白进行分离,组蛋白条带清晰可见(见图3b),提取效果理想。

**图3:**
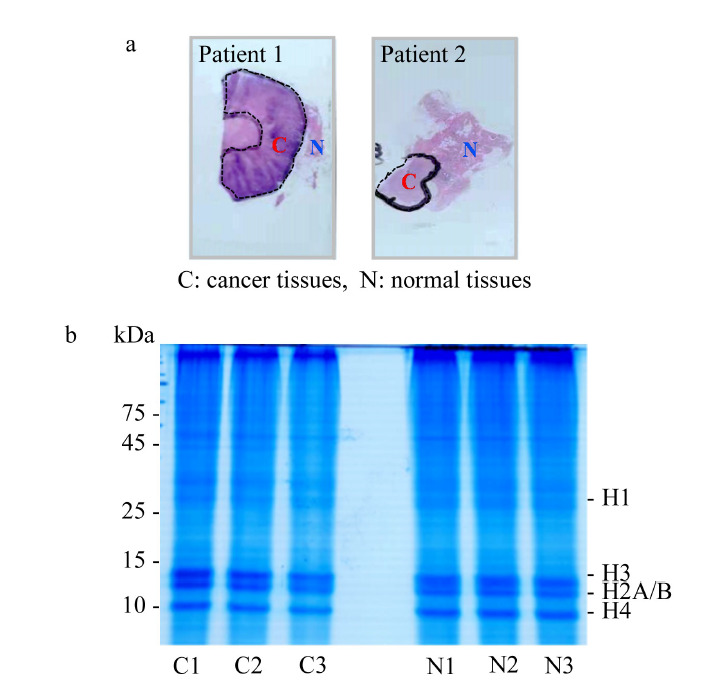
(a)石蜡包埋乳腺癌组织切片中癌与癌旁正常组织的选取及(b)组蛋白的分离

2.2.2 组蛋白翻译后修饰的定性鉴定

将组蛋白条带分别切下,经胶内酶解后,进行质谱分析,通过Proteome Discoverer软件对数据进行检索,组蛋白H3和H4翻译后修饰的初步鉴定结果见图4。在浸润性癌组织中,除了鉴定到常见的赖氨酸甲基化、乙酰化修饰,我们也发现了一些低丰度的修饰如赖氨酸巴豆酰化、2-羟基异丁酰化等。

**图4:**
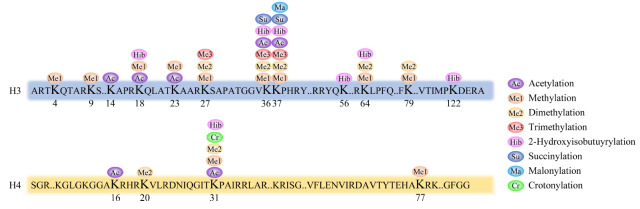
癌组织中组蛋白翻译后修饰的鉴定

2.2.3 组蛋白翻译后修饰的定量分析

将组蛋白条带全部切下,经组蛋白胶内丙酸酐化学衍生化、胶内酶解、多肽N端丙酸酐化学衍生化后,进行HPLC-MS/MS分析,采用EpiProfile软件对组蛋白翻译后修饰进行定量分析。为了考察数据稳定性与重复性,本研究分别进行了鉴定修饰多肽数目比较、相关性分析、主成分分析(PCA)等生物信息学分析。通过对鉴定修饰多肽数目进行统计(见图5a),各样品鉴定的不同修饰形式的多肽数目均在100以上,具有较好的鉴定信息。根据相关性分析结果(见图5b),样品内3次重复的皮尔逊相关系数在0.8以上,具有较强的相关性。

**图5:**
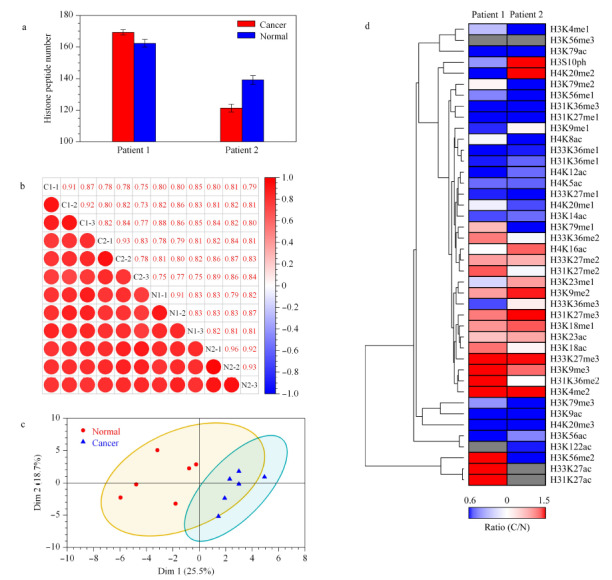
癌组织和癌旁正常组织中组蛋白翻译后修饰的定量分析

而两例Her2+患者浸润性癌和癌旁组织样品之间的相关性约在0.7以上,也具有较强相关性,这表明实验整体上具有较好的稳定性。根据主成分降维分析结果(见图5c),浸润性癌和癌旁正常组织之间组蛋白翻译后修饰定量结果存在一定的差异性,这表明组蛋白修饰具有一定区分癌和癌旁正常组织的评价潜力。通过对组蛋白修饰定量结果的聚类分析(见图5d),发现很多HPTMs位点的丰度在两例Her2+患者乳腺浸润性癌和癌旁正常组织中具有明显的差异,而两例样本之间的差异性规律是相近的,其中包括一些与转录激活或抑制密切相关的组蛋白修饰,如H3K9me3、H3K9ac、H3K27me3等,这些修饰的不同暗示Her2+患者乳腺浸润性癌和癌旁正常组织中的表观调控存在差异。这些一致性的组蛋白修饰规律可能与Her2+乳腺浸润性癌的特征具有一定联系。此外,我们特别注意到浸润性癌组织中H4K20me3均显著下调,这也与文献^[17]^报道一致,H4K20me3下调被认为是人类癌症的一个标志,也是包括乳腺癌在内的许多类型癌症的潜在预后标志,而组蛋白H4K20甲基转移酶SUV420H2也被认为是癌症潜在的治疗靶标^[18]^。我们也发现,两例样本之间也存在一些不同的规律,如H3S10磷酸化修饰在两例患者乳腺浸润性癌和癌旁正常组织中呈相反规律,这可能与患者个体或成瘤的差异有一定关系。

## 3 结论

本研究建立了一种基于高效液相色谱-串联质谱的石蜡包埋临床样本HPTMs提取分离、定量分析新方法,并结合乳腺癌的实际样本,尝试分析了浸润性癌和癌旁正常组织的HPTMs。从结果来看,这一方法在临床肿瘤样本分析中具有应用潜力,这方面工作的深入开展有利于从大量石蜡包埋组织中,探索基于组蛋白修饰的肿瘤标志物,为肿瘤临床治疗靶点的寻找、转移复发风险的预测提供有益的信息。
